# Monitoring a cohort of trainees: changes over time and associations between health literacy, health behaviour and health

**DOI:** 10.1186/s12995-023-00387-1

**Published:** 2023-08-29

**Authors:** Peter Koch, Jan Felix Kersten, Albert Nienhaus

**Affiliations:** 1 Competence Center for Epidemiology and Health Services Research for Healthcare Professionals (CVcare), Institute for Health Services Research in Dermatology and Nursing (IVDP), University Medical Center Hamburg-Eppendorf (UKE), Hamburg, Germany; 2 Department for Occupational Medicine, Hazardous Substances and Health Sciences (AGG), German Social Accident Insurance for the Health and Welfare Services (BGW), Hamburg, Germany

**Keywords:** Health literacy, Prevention, Adolescents, Occupational health, Psychological well being

## Abstract

**Background:**

For many entrants, the initial stages of professional training are a challenge. Demands at work can lead to new physical and psychological stress, as well as new social requirements. These new requirements can influence the health behaviour and the state of health of young employees. In recent years, there have been many studies on health literacy (HL). HL represents resources and potentials that allow individuals to achieve improved control of their health and of factors that influence health. Thus, HL can influence both well-being and health. In the present study, the health of trainees in different branches (health and welfare services, office, sales, technology) is monitored over time ending in the period of the COVID-19 pandemic. Furthermore, the association between health literacy and health or health behaviour has been examined.

**Methods:**

In 2017/18, a baseline survey (T0) was performed on trainees in various sectors (office, sales, teaching, nursing and social welfare, engineering, hairdressers), who had been contacted through vocational colleges in four federal states in north Germany. The trainees were surveyed again in the in the first year after training in 2021 (T3). Demographic data were collected, as well as information on health literacy (HLS-EU-16), health behaviour (physical exercise, nutrition, smoking and alcohol) and state of health (BMI, psychological well-being and subjective state of health). Recognition, satisfaction at work and thoughts of leaving the profession were surveyed with the Copenhagen Psychosocial Questionnaire (COPSOQ). Statistical analysis was performed with tests for paired samples and multivariate regression analysis in SPSS 26.

**Results:**

129 data sets were evaluated, with a follow-up rate of 10.2%. 85% of the trainees were female. The mean age at follow-up was 25.6 years. 56% were employed in the health service or social welfare. 35% worked in the office, sales or engineering. At T3, 47% of the employees exhibited limited health literacy, 67% low levels of exercise and 30% risky alcohol use. 42% exhibited overweight and 42% poor psychological well-being. An association between health literacy (HL) and psychological well-being was only observed in cross-section (HL inadequate vs. HL adequate OR: 3.2 95% CI: 1.07–9.49, p = 0.037). The odds ratio relative to subjective state of health was also increased, although the association was not statistically significant (HL inadequate vs. HL adequate OR: 2.7 95% CI: 0.72–9.78, p = 0.143). In the sector for health service and social welfare, there was statistically significant deterioration over time in all COPSOQ variables (recognition, satisfaction at work, thoughts of leaving the profession).

**Conclusions:**

For a group of trainees in the first year of work during the covid-19 epidemic, the present findings show that there is a need to prevent risky health behaviour, overweight and poor psychological well-being. Health literacy was shown to be a modifiable parameter, that apparently is associated in cross – but not in longitudinal section with the health of young employees. It would appear to be reasonable to modify developing health literacy in the setting of work and school.

## Introduction

In Germany, there are about 1.3 million trainees who are undergoing training in companies that offer training in vocational colleges. About a quarter of contracts for professional training are prematurely cancelled each year [1]. Many people starting professional training find this challenging. These stressors are experienced by young people at risk, who are still in the process of developing into adults and who generally do not consider that unhealthy behaviour can have adverse effects on their health. Moreover, they have little awareness of health protection or protection at work [2]. These new demands may influence the health behaviour and state of health of young employees. Studies have demonstrated the unfavourable effects of these demands on e.g. eating habits and the extent of physical activity [3]. In addition, health problems, such as pain in the back or headache have been associated with activities at work [4]. In view of this vulnerable phase of life and the lack of trained employees in some sectors, it is important to monitor the health and health behaviour of young people at their workplace, in order to identify measures to support health and prevention for each workplace. These measures can have synergistic effects on the individual health of young workers on the one hand and on the prevention of a shortage of skilled workers on the other.

### Theoretical background of health literacy

The term health literacy (HL) is introduced in the 1970 [5]. It is fundamental to distinguish HL from literacy in general. While literacy refers to the ability to read and write, HL is not only the ability to use and understand words and numbers in a medical context. The term HL also encompasses the use of different abilities, such as reading and acting upon written health information, communicating needs to health professionals, and understanding health instructions [6].

In recent years, there have been many research studies on health literacy (HL). On the basis of a systematic review Sørensen et al. developed a definition of HL: “Health literacy is linked to literacy and entails people’s knowledge, motivation and competences to access, understand, appraise and apply health information in order to make judgments and take decisions in everyday life concerning healthcare, disease prevention and health promotion to maintain or improve quality of life during the life course” [7]. So HL represents the resources and potentials that allow individuals to achieve more control over their health, as well as over factors that influence their health [8].

### Research results on health literacy

International studies have demonstrated HL deficits in many countries [9]. In Germany, more than half the population (59%) exhibit deficits in HL [10].Associations between HL and health indicators have been demonstrated in both the general population and in specific subgroups. These include socio-economically disadvantaged adults, patients, nurses and their children as well as adolescents [11–16]. As health behaviour is apparently a mediator between HL and health, there is also published evidence for an association between HL and health behaviour [17–20]. In their systematic review including studies investigating adolescent HL and health behaviour Fleary et al. found a broad range of different health behaviour components in the original studies e.g. alcohol use, tobacco use, medical adherence, health-related information seeking, risky sexual behaviour, physical exercise, nutrition etc. [17]. In summary the relationship between HL and health behaviour is clearer in studies of adults than of adolescents [17].

Within the setting of the workplace, a person with a good HL can consider how their health is related to their profession and can take appropriate action to help to reduce accidents at work and occupational diseases [21, 22]. This has led to the development of current concepts, such as the National Action Plan for Health Literacy, which are intended to stimulate HL in the workplace and in the educational system [22, 23].

The present study investigates the changes during time of HL, health behaviour and health within a cohort of trainees (i). The study will also analyse if workers with an inadequate HL have a greater risk for an unfavourable health behaviour (ii) and if workers with an inadequate HL have a greater risk for an unfavourable health status (iii).

## Methods

The present study presents longitudinal data for a study cohort of trainees, as observed during and after their training period [24]. These are trainees from eleven different qualified professions, summarised in two groups: firstly, office, technology and sales (office administrator, retail and wholesale sales, industrial business management, retail salesperson, plant manager for plumbing, heating and air conditioning, electrician for plant technology and building technology) and secondly, health service and social welfare (geriatric nurse, nurse or hospital nurse, medical assistant, childcare worker, hairdresser). All occupational groups of the latter group traditionally belong to the group of insured persons of the German statutory accident insurance.

### Data collection

The baseline survey for the study (T0) was performed at the end of 2017 or the beginning of 2018. The follow-up T1 was performed in the middle of training, in March 2019. Follow-up T2 was performed at the end of training in July 2020. Follow-up T3 was performed during the first year of employment in October 2021. The overall follow-up was about 4 years. At the start of the study, we carried out an Internet search for all vocational colleges offering the relevant training courses in the northern federal states of Germany (Schleswig-Holstein, Bremen, Lower Saxony and Mecklenburg-Western Pomerania), which we then contacted. We had no approval from the school authorities for the federal state of Hamburg. We were awarded a favourable ethics vote for the study by Hamburg Medical Association (PV5670). Out of 321 identified vocational collages 47 participated in the study (response 14.6%). Further details can be found in the corresponding publication of the baseline survey [24].

Of the 321 identified vocational colleges, 46 agreed to participate in the study (response rate 14.6%). In October 2017, a total of 5052 trainees were invited to participate in the study and 1797 of these returned the questionnaire to the study centre (response rate 35.5%) In the first year of employment after completion of training (T3), a printed form was sent to the private addresses of 1569 trainees who had consented to a follow-up survey. A total of 160 questionnaires were returned to the study centre (follow-up rate 10.2%) from which 31 cases documented a discontinuation of training due to different reasons. Finally, 129 cases were included in the analysis sample.

### Operationalization of variables

The baseline survey incorporated sociodemographic data on age, gender, country of birth, nationality and highest academic qualification. HL was surveyed with the validated short questionnaire HLS-EU-Q16 [25].

The four-step answer categories were dichotomised and a cumulative score of 0 to 16 points (P) was calculated. On this basis, we applied classification into three HL levels: adequate (13–16 P), problematical (9–12 P) and inadequate (0–8 P) [25]. If values were missing for more than two items, the cumulative score was rated as “missing”. Reliability analysis of the original items of the HL scale provided a Cronbach’s alpha of 0.88.

#### Health behaviour

The frequency of physical exercise was surveyed on the basis of five categories (none/<1 h per week/1 > 2 h per week/2 > 4 h per week/>= 4 h per week) [26]. In addition, smoking habits [27], as well as alcohol use with the Audit C questionnaire [28], and nutritional behaviour were assessed [29]. A nutritional score was calculated on the basis of 15 food items. On the basis of a 6-step frequency of consumption (daily to never), from 0 to 2 points (abnormal, normal, optimal frequency of consumption) were awarded for each of the surveyed groups of food. A score (range 0 to 30) was calculated from the sum of the points. This was used to form categories of nutritional patterns, ranging from optimal nutritional pattern (16–30 points), normal nutritional pattern (13–15 points) to unfavourable nutritional pattern (0–12 points).

#### State of health

A total of four indicators of the state of health were surveyed. Aside from the BMI, the subjective state of health was assessed with a one item 5-step evaluation (excellent/very good/good/less good/poor) [3] and dichotomised as follows: *good*: excellent/very good/good and *poor*: less good/poor. Various diseases with a medical diagnosis within the previous twelve months were surveyed from the items in the work ability index [30]. Psychological well-being was assessed from the WHO-5 Index and reached a good internal consistency with a Cronbach’s alpha of 0,87 [31]. The dichotomisation of the WHO-5 index was based on the published cut-off scale value (poor psychological well-being: < 13).

#### Workplace-related psychosocial factors

On the basis of the COPSOQ questionnaire, recognition (1 Item), satisfaction at work (6 Items, Cronbach’s alpha : 0.85) and thoughts of leaving the profession (1 Item) were assessed [32].

### Statistical analysis

For categorical data, Fisher’s exact test was used for comparing groups. For normally distributed metric data, groups were compared with the t test. The Mann Whitney U test (2 samples) and Kruskal-Wallis test (k samples) were used for metric data that was not normally distributed. In order to examine changes over time in HL, health indicators and health behaviour tests for dependent samples were calculated. Nominal data were then examined with the McNemar test, data without normal distribution with the Wilcoxon test and normally distributed data with the t test.

Hypothesis testing in longitudinal design was performed by multivariate logistic regression. As age and sex represent health disparities in populations, these characteristics were inserted as adjustment variables into the multivariate models [33, 34].The extent to which health literacy at T0 has an influence on the indicators of health and health behaviour at T3 was examined. Effect estimates were reported with 95% confidence intervals. The level of significance was p < 0.05. Statistical analysis was carried out with SPSS 26 (IBM Corp., Armonk, NY, USA).

## Results

### Description of the cohort

At the time point of the fourth survey, 160 questionnaires were received in the study centre at T3 (follow-up rate: 10.2%). 31 persons reported that they no longer worked in the profession which they had studied; thus 129 persons were included in the analysis. 65% had been trained to work in the health service or in social welfare, 35% had been trained in office, sales or technological professions (Table 1). The mean age was 25.6 years, with an interquartile range of 3 years. 85% of participants were female and 97% were German citizens. At the time of the baseline survey, the subjects most often had a secondary school leaving exam [Realschule] (47%). 34% had taken Abitur [A-Levels]; 16% had taken a leaving exam from a technical college and 3% a leaving exam for basic secondary school [Hauptschule].

**Table 1:** Description of the cohort (N = 129)

	**Health and Welfare Services**	Office, Sales, Technology	Total Group	p
	**n = 84 (65%)**	**n = 45 (35%)**	n = 129 (100%)	
**Age in years (first year of employment)**				**0.023**
mean (standard deviation):	26.3 (6.1)	24.5 (2.8)	25.6 (5.2)	
minimum, Maximum:	20, 54	20, 35	20, 54	
interquartile range:	4	2	3	
**Sex**				**< 0.001**
female	81 (96%)	29 (64%)	110 (85%)	
**Nationality**				0.674
German	81 (96%)	44 (98%)	125 (97%)	
other	3 (4%)	1 (2%)	4 (3%)	
**School leaving exam**				**0.020**
basic secondary school	3 (4%)	1 (2%)	4 (3%)	
secondary school	46 (54%)	15 (33%)	61 (47%)	
technical college	15 (18%)	5 (11%)	20 (16%)	
abitur [A-levels]	20 (24%)	24 (54%)	44 (34%)	

#### Changes over time in health literacy (HL), indicators of health behaviour and health COPSOQ variables

**Fig. 1:**
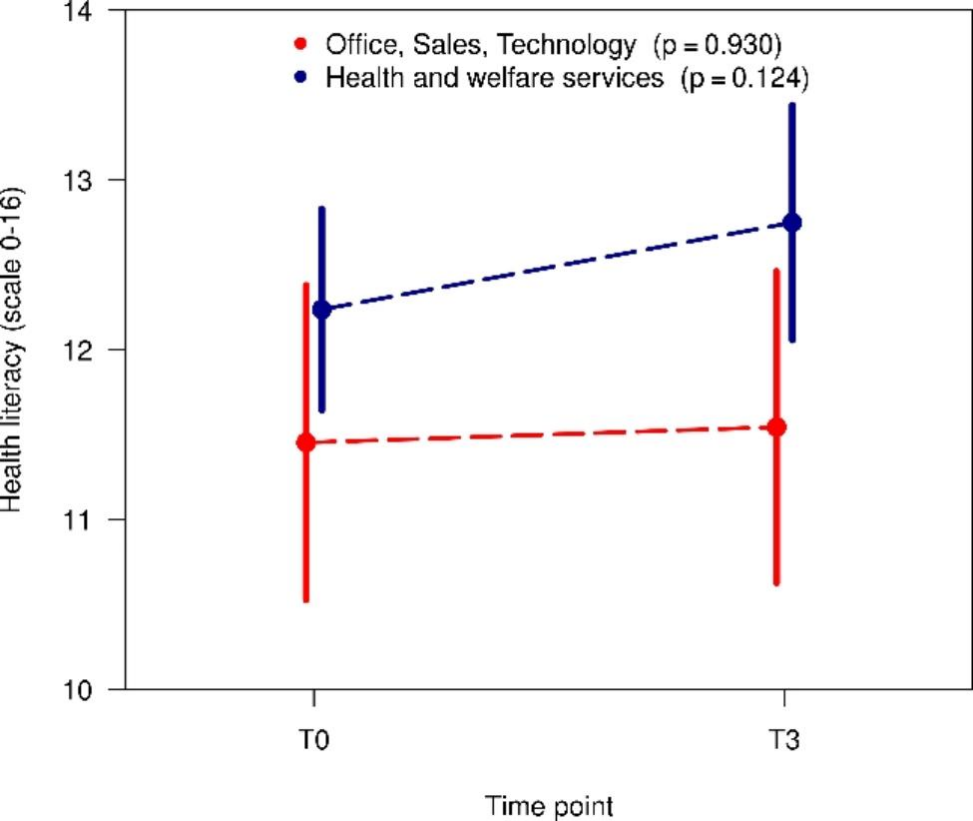
Changes over time in health literacy in the sectors (mean and 95% confidence interval)

**Fig. 2:**
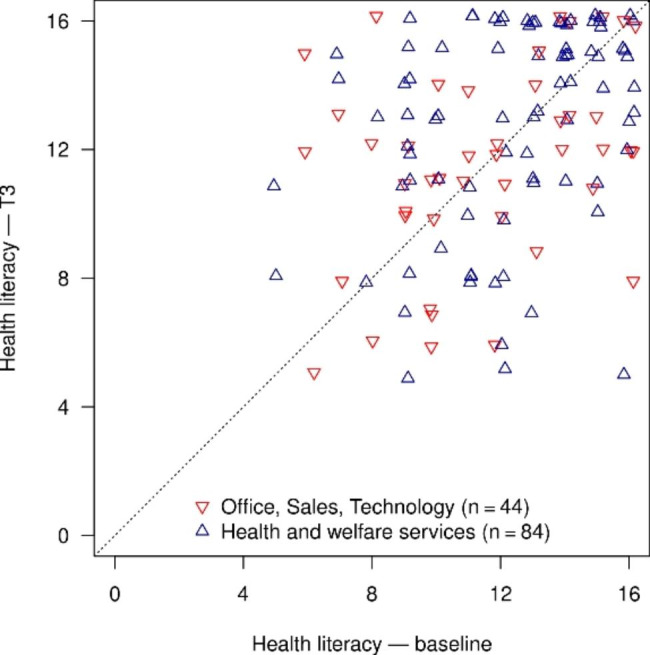
Scatter diagram of health literacy at T0 and T3

The changes over time in HL, health behaviour and indicators of state of health are presented in Table 2. For health literacy, there was a slight increase in the mean value over time (T0: 12.0 vs. T3: 12.3, p = 0.223). Referring to the categorical variable of HL the proportion of limited HL (problematic or inadequate HL) decreases from 53 to 47%. Employees in health and welfare services started at T0 with a greater mean value than employees in other sectors (T0: 12.2 vs. 11.5) and also had a higher mean value at T3 (T3: 12.8 vs. 12.2) (Fig. 1). The increases in HL in the two groups of sectors are not significant. The individual changes over time in HL are also heterogeneous. Both increases (49%) and decreases (38%) of the HL score in the two sector groups were recorded; in 13%, the value was unchanged (Fig. 2).

For the variables in health behaviour (Table 2), the only statistically significant difference was for risky alcohol use: the proportion of subjects with risks alcohol use fell from 41 to 30% (p = 0.011). This reduction was statistically significant in both groups of sectors. The proportions for the other health behaviour indicators at time point T3 were 51% for unfavourable nutrition, 67% for low physical exercise and 26% for smoking.

For health indicators, there was a statistically significant increase in mean BMI (T0: 24.3 vs. T3: 25.3, p < 0.001). This statistically significant increase was also observed in both groups of sectors. For the total sample the proportion of overweight/adipositas increased from 36 to 42%. Over this period, the mean subjective state of health decreased slightly (T0: 3.4 vs. T3: 3.2, p = 0.123), the proportion of a less good/poor subjective state of health increased from 13 to 17%. In addition, psychological well-being decreased over time (T0: 13.9 vs. T3: 13.2, p = 0.105), the proportion of low psychological well-being increased from 35 to 42%. Neither change over time was statistically significant. At both time points, the prevalence values for medically diagnosed diseases lay between 9% and 23%. For cardiovascular diseases, the prevalence at T0 and T3 was 5%. There were no statistically significance differences observed over time in any type of disease.

**Fig. 3:**
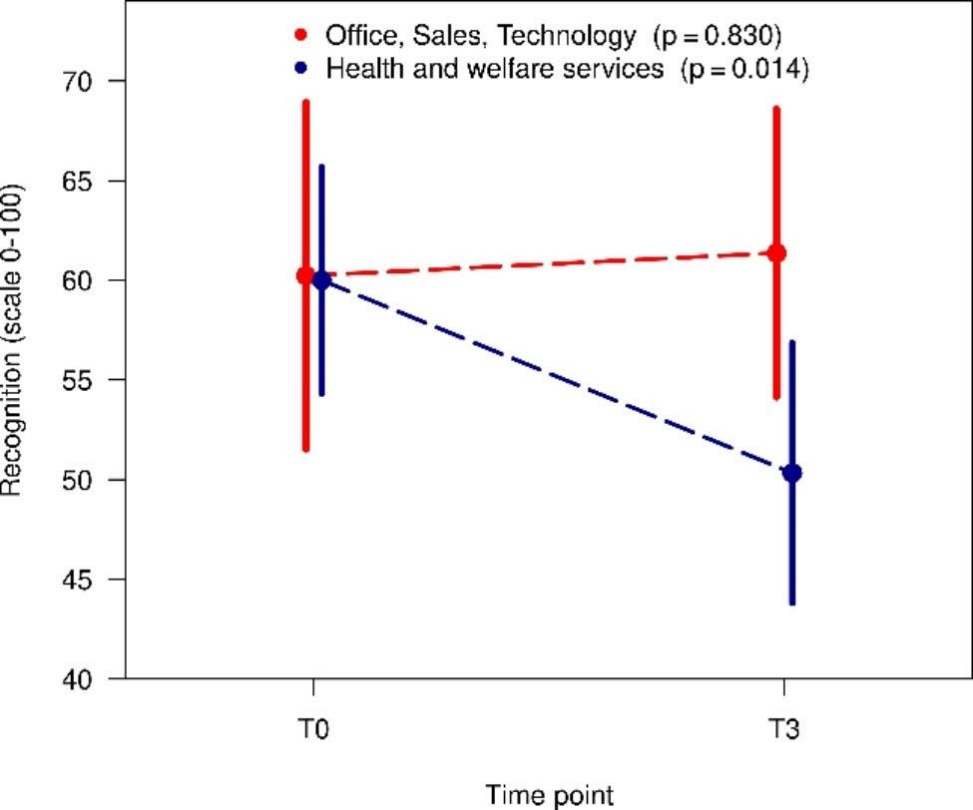
Changes over time in recognition in different sectors (mean and 95% confidence interval)

**Fig. 4:**
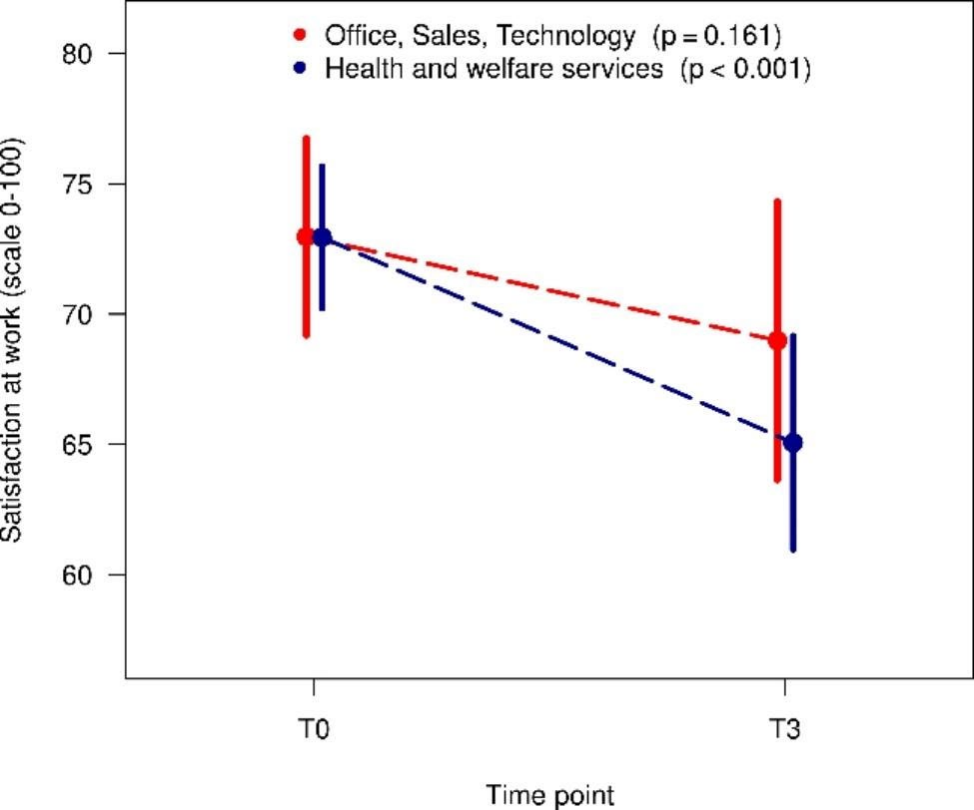
Changes over time in satisfaction at work in different sectors (mean and 95% confidence interval)

**Fig. 5:**
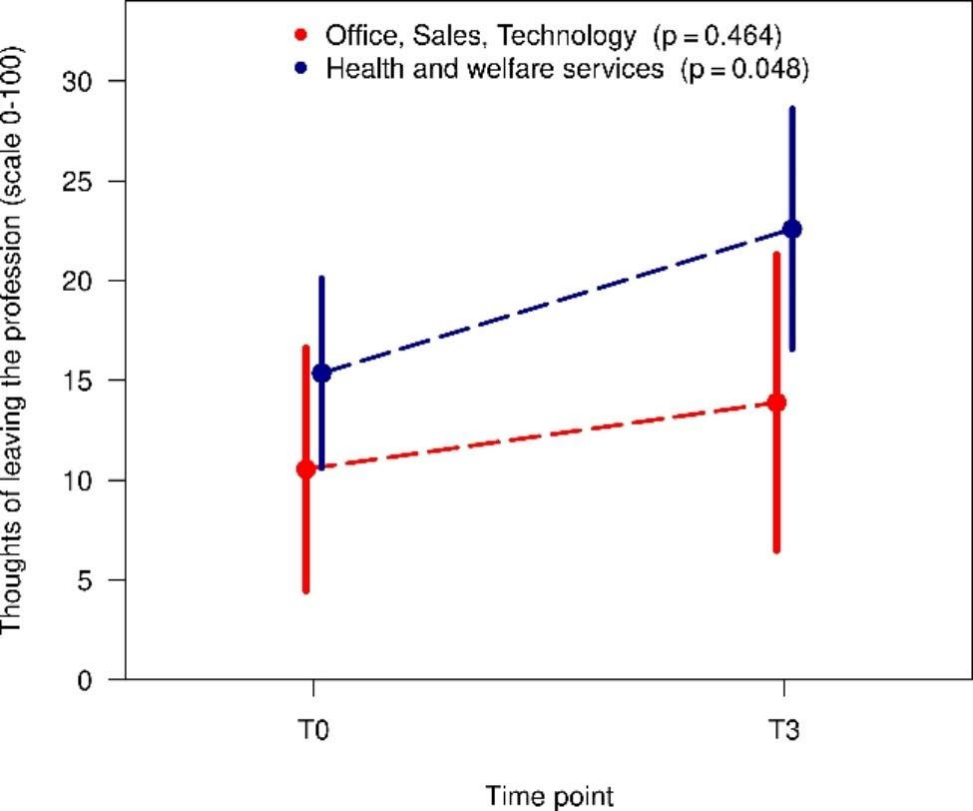
Changes over time in thoughts of leaving the profession in different sectors (mean and 95% confidence intervals)

In all three cases, there was a deterioration in COPSOQ variables. In the whole group, there was a reduction in mean recognition from 60.1 to 54.4. For the sector health and welfare services, this reduction was statistically significant (T0: 60.0 vs. T3: 50.3, p = 0.014) (Fig. 3) In Figs. 4 and 5, it is clear that the statistically significant reduction in the mean for the whole group is solely due to the subgroup health and welfare services (satisfaction at work T0: 72.9 vs. T3: 65.1, p < 0.001, thoughts of leaving the profession T0: 15.4 vs. T3: 22.6, p = 0.048).

Table 3 portrays the time course of the work-related parameters within the period of training and compares these with changes between the start of training to the first year of employment (T0- T3), Table 3 shows the changes over time from T0 to T2 within the two groups of sectors (n = 189). This shows that there is no significant reduction within the two groups for the mean value of recognition and thoughts of leaving the profession, but a significant reduction in satisfaction at work (office/sales/technology p = 0.010, health and welfare services p = 0.025).

As regards the comparison between the two groups of sectors at the time point T3, it could be observed that employees in health and welfare services exhibited greater HL (x̅: 12.8 vs. 11.6, p = 0.044), lower recognition (x̅: 50.3 vs. 61.7, p = 0.022), poorer subjective state of health (x̅: 3.1 vs. 3.5, p = 0.015), as well as more frequent diseases of the respiratory tract or hormonal diseases (18% vs. 5%, p = 0.035 and 19% vs. 5%, p = 0.024, respectively) (data not shown in the table).

**Table 2:** Changes over time in health literacy, indicators for health behaviour and health (N = 129)

Variable		T0 x̅ ^a^ (SD ^b^), %	T3 x̅ ^a^ (SD ^b^), %	p
**Health literacy**	scale (0–16)	12.0 (2.9)	12.3 (3.2)	0.223
	adequate problematical inadequate	47% 42% 11%	53% 30% 17%	
**Nutrition**	scale (0–30)	13.1 (3.2)	12.8 (3.7)	0.403
	favourable normal unfavourable	22% 36% 42%	24% 25% 51%	
**Fast food**	more than once per week	9%	13%	0.359
**Smoking**	yes	28%	26%	0.804
**Physical exercise < 2 h/week**	less than 2 h/week	59%	67%	0.124
**Risky alcohol use**	yes	41%	30%	**0.011**
**BMI**	continuous	24.3 (5.1)	25.3 (5.6)	**< 0.001**
	underweight normal weight overweight adipositas	4% 60% 21% 15%	2% 56% 23% 19%	
**Subjective state of health**	scale (1–5)	3.4 (0.82)	3.2 (0.84)	0.123
	excellent/very good good less good/poor	40% 47% 13%	37% 46% 17%	
**Medically diagnosed diseases (previous 12 months)**	musculoskeletal system	22%	23%	1
	skin	17%	15%	0.815
	respiratory tract	17%	13%	0.523
	psyche	18%	18%	1
	Neurological	14%	16%	0.832
	digestive system	12%	9%	0.648
	hormonal	11%	14%	0.424
	cardiovascular	5%	5%	1
**Psychological well-being**	scale (0–25)	13.9 (4.5)	13.2 (5.1)	0.105
	poor psychological well-being (< 13 points)	35%	42%	

^a^mean, ^b^standard deviation

**Table 3:** Time course of COPSOQ variables from start of training at T0 to completion of training at T2 (n = 189)

Variable	Group of sectors	T0 x̅ ^a^ (SD ^b^), %	T2 x̅ ^a^ (SD ^b^), %	p
**Recognition**	office/sales/technology	55.8 (27.4)	49.0 (27.8)	0.065
	health and welfare services	56.2 (23.8)	59.4 (24.7)	0.346
**Satisfaction at work**	office/sales/technology	73.1 (14.0)	68.3 (16.7)	0.010
	health and welfare services	71.7 (11.8)	68.3 (16.7)	0.025
**Thoughts of leaving the profession**	office/sales/technology	12.6 (22.6)	11.1 (15.9)	0.469
	health and welfare services	13.9 (20.4)	16.0 (22.9)	0.363

^a^mean, ^b^standard deviation

#### Association between health literacy and indicators of health behaviour and health

No associations were found between HL T0 and indicators of health behaviour T3; this applied both longitudinally and in cross-section (T3). Two additional longitudinal analyses on this association with a reduced sample size (HL T1/T2 and health behaviour T3) also showed no associations (data not shown).

No longitudinal associations were found between HL T0 and indicators of health T3 (subjective state of health and psychological well-being). Two additional longitudinal analyses on this association with a reduced sample size (HL T1/T2 and indicators of health T3) also showed no associations (data not shown). Cross-sectional bivariate analysis showed no statistically significant differences in the median of state of health (T3) across the HL (T3) categories (HL adequate: 3, HL problematic: 3, HL inadequate: 3, p = 0,169). In contrast, there was a cross-sectional association between HL (T3) and subjective state of health (T3) (Table 4). In comparison to persons with adequate HL, persons with inadequate HL exhibited an increased odds ratio of 2.7 (95% CI: 0.72–9.78, p = 0.143), although this was not statistically significant. For each reported disease in the last 12 months, the risk increased by 60% for an less good subjective health status (OR: 1.6, 95% CI: 1.15–2.21, p = 0.005).

For HL (T3) and psychological well-being (T3), statistically significant differences in the median were found (HL adequate: 15, HL problematic: 13, HL inadequate: 11, p = 0,036). In the multivariate model a statistically significant cross-sectional association was also found (Table 5). Thus, in comparison to persons with adequate HL, persons with inadequate health literacy exhibited an odds ratio that was increased by 220% (3.2 95% CI: 1.07–9.49, p = 0.037). Persons with a problematical HL also exhibit an increased odds ratio, although this was not statistically significant (OR: 1.8 95% CI: 0.76–4.42, p = 0.182). For women, there was an increased odds ratio for poor psychological well-being relative to men which was statistically not significant (OR: 2.8 95% CI: 0.80–9.67, p = 0.187).

**Table 4:** Multivariate logistical regression: health literacy (T3) on less good subjective state of health (T3)

Variable	OR (95% CI) univariate	p	OR* (95% CI) multivariate	p
health literacy adequate (13–16 points)	1	-	1	-
health literacy problematical (9–12 points)	2.0 (0.68–5.85)	0.206	2.2 (0.70–7.17)	0.174
health literacy inadequate (0–8 points)	3.0 (0.90–9.96)	0.073	2.7 (0.72–9.78)	0.143
Sum of diseases last 12 month (per one disease)	1.7 (1.24–2.26)	0.001	1.6 (1.15–2.21)	0.005
sex: female vs. male	4.3 (0.55–34.41)	0.164	3.4 (0.41–28.71)	0.259

Less good subjective state of health: 17%, missing data 2%, Nagelkerkes r^2^: 0.22, *adjusted for age

**Table 5:** Multivariate logistic regression: health literacy (T3) on psychological well-being (T3)

Variable	OR (95% CI) univariate	p	OR* (95% CI) multivariate	p
health literacy adequate (13–16 points)	1	-	1	-
health literacy problematical (9–12 points)	1.7 (0.75–3.75)	0.208	1.8 (0.76–4.42)	0.182
health literacy inadequate (0–8 points)	3.2 (1.15–8.76)	0.025	3.2 (1.07–9.49)	0.037
Sum of diseases last 12 month (per one disease)	1.6 (1.23–2.12)	0.001	1.6 (1.17–2.09)	0.003
sex: female vs. male	3.2 (0.99–10.19)	0.052	2.8 (0.80–9.67)	0.187

Poor psychological well-being: 42%, missing data 2%, Nagelkerkes r^2^: 0.23, *adjusted for age

## Discussion

The present investigation observes a cohort of trainees, from the start of training till the first year of employment. The proportion of limited HL (problematic or inadequate HL) was 47%. As regards the health behaviour, there were high prevalence values for unfavourable nutrition (51%), smoking (26%), low physical exercise (67%) and risky alcohol use (30%). As regards health indicators, 42% of persons were overweight, 17% reported poor subjective state of health and 42% of persons exhibited low psychological well-being. No longitudinal associations were found between HL and health behaviour. A statistical significant cross-sectional odds ratio of 3.2 was observed for the association with psychological well-being.

### Changes over time

For HL, there was a slight increase in the mean value over time. This was particularly marked in the health and welfare services sector group, but was not statistically significant. The level of HL at T0 was higher for employees in health and welfare services than for persons from the sector office/sales/technology – presumably due to selection. The reason is evidently that the various courses for training in the health and welfare services gradually lead to greater increases than in the comparative group; these increases are statistically significant at time point T3. There were nevertheless heterogeneous changes in HL over time. Thus, a decrease in the HL of at least one point was observed in 38% of participants. This may indicate a bias due to a faulty subjective assessment at T0.

The observed prevalence of limited (problematic or inadequate) HL was 47%, which is less than the values of 53% and 49%, respectively, found in the baseline and follow-up T1 publication and is lower than data from the German speaking area [24, 35]. In the recently performed survey in the adult German population, this value was 59% [10]; in 2015, Jordan & Hoebel observed a prevalence of 44% [36]. For younger subjects, the proportion of limited HL has been reported to be 58% (15-year old Austrians) or 69% (students in a German vocational college for health) [25, 37].

A statistically significant reduction in *risky alcohol use* was observed in both groups of sectors and represents a continuation of the trend observed in the T1 publication of the cohort [35]. Nevertheless, the prevalence value of 30% was clearly above the comparative German data for the age group of 18 to 29 years, where the gender-specific values were 14% for men and 18% for women [38]. The *increase in BMI* over the time in this age group is consistent with the data from German health reports, even though the value of 25.3 found in this study was above the reference values in the official statistics (20–24 years: 23.5, 25–30 years: 24.5) (39). A possible explanation for this increase could be the restrictions on activities in the two lockdown phases in Germany during the observation period of the study (1st lockdown: March 2020 to May 2020, 2nd lockdown: December 2020 to May 2021) However, due to the lack of a comparison cohort, this explanation is purely hypothetical. The prevalence of *poor subjective state of health* was 17%, which is clearly greater than the comparable values in the GEDA 2014/2015 EHIS survey, where the gender-specific prevalence were between 2.5% (women) and 1% (men) in the age group of 18–29 years [40]. In addition, a large section of the subjects (42%) reported *poor psychological well-being* at T3. It is certainly possible that the generally high values for unfavourable health behaviour (unfavourable nutrition: 51%, smoking: 26%, low physical exercise: 67%, risky alcohol use: 30%) and poor psychological and physical health are at least to some extent consequences of the stress from the covid-19 pandemic in Germany, with the restrictions to social contact in 2021, and should be regarded as a period effects of the pandemic.

There were striking changes from T0 to T3 in the workplace-related variables of recognition, satisfaction at work and commitment. These changes depended on the sector, namely that in the health and welfare services sector there were statistically significant deteriorations over time in all three parameters. On the one hand, the mean for recognition tended to increase during the training period (T0-T2) for health and welfare services, with a slight decrease in commitment (neither statistically significant). On the other hand, for the period T0-T3, the values for recognition, commitment and satisfaction at work deteriorated in this group of sectors. This indicates that for the group health and welfare services, the transition from the training period into actual employment is more difficult with respect to the workplace parameters examined here. It may also indicate that the period effect from the covid-19 pandemic is more marked for employees in social sectors than in other sectors. Daily care of patients and clients in the context of a pandemic is more stressful, due to the inevitable contact restrictions, distancing and the relatively high risk of infection. This may all cause entrants to have doubts about their choice of profession. It is difficult to assess the specific mean values we found for the COPSOQ scales, as the groups include different subsectors.

### HL and indicators of health behaviour and health

No associations were observed between HL at T0/T1/T2 and the various indicators of health behaviour at T3. An alternative approach was to consider that HL might act immediately on health behaviour, so that HL at T3 should be compared with health behaviour at T3 – but here too no associations were found. This negative result is consistent with the lack of association between HL and health behaviour in the T1 publication [35]. On the other hand, this lack of association between HL and health behaviour is, to some extent, incompatible with the results of previous studies. In a systematic review, Fleary et al., reported that 13 of 17 studies on adolescents found statistically significant associations between HL and health behaviour [17]. A German study on adolescents with limited HL employed the long version of our questionnaire (HLS-EU-Q47) and found no associations with tobacco and alcohol use, but associations with nutrition and exercise. Moreover, a study with 15-year old Austrian adolescents failed to find an association between two out of three indicators of health behaviour (alcohol use and smoking; the association with exercise was weak (r = 0.14)) [25]. On the other hand, a systematic review of interventions in adults found a clear association between HL and the outcome health behaviour [41]. Thus, published reports on this association are inconsistent. In the present study; the lack of association between HL and health behaviour may be because our subjects wrongly assessed their HL, due to their relative youth. This would indeed be consistent with the decreases in HL over time in our study. Another possible explanation would be that the subjects accept the risk of unhealthy behaviour, even though they know better. In focus groups, Joseph et al. considered that HL provided an instrument for making well informed decisions. Nevertheless, the participants admitted that they sometimes failed to exploit these insights by adopting risky behaviour [42]. Another possible explanation is as follows. In a mediation analysis, one study with the Chinese version of the 47 item HLS-EU-Q47 concluded that only the subscale *Use Information* had an indirect effect on health through health behaviour [43]. It seems plausible that the content of this subscale may be more directly related to health behaviour than are the subscales *Find Information*, *Understand Information* or *Assess Information*. We were unable to replicate this analysis in our study, as we used an item-reduced version of the HLS-EU-Q16.

For psychological well-being, an increased cross-sectional odds ratio of 3.2 was found for inadequate HL and of 1.8 for problematical HL (not statistically significant). This confirms the trend in the results in the T1 publication [35]. This result was also confirmed by Björnsen et al. in a study on adolescents [44]. In a cross-sectional study with Norwegian (male and female) adolescents aged 15 to 21 years, it was found that high mental HL was associated with high psychological well-being. This was confirmed in additional cross-sectional studies with adults. Zhang et al. observed this association in a population-related study in Hong Kong, analogous results were reported by Fiedler et al. in German industry managers and by Amoah et al. in a population-related sample in Ghana [16, 45, 46].

There were cross-sectional associations between HL and subjective state of health, although the estimates were not statistically significant. Employees with inadequate HL have a 2.7-fold greater probability of lower subjective state of health than those with adequate HL; there is a 2.2-fold difference for persons with problematical HL. This result confirms the statistically significant results in the T1 publication [35]. Little has been published on the effect of HL on subjective health in this target group. Two studies with vocational students show that young adults with high HL also have better subjective health than students with low HL [47, 48]. In a systematic review, Sansom-Daly et al., concluded that there had been very few studies in adolescents on the association between HL and various health indicators and that the results of these studies were inconsistent [19]. On the other hand, a large population-related study in China demonstrated unambiguous associations between HL and subjective state of health [49].

The associations were only found in cross-section and this indicates that there is a direct temporal relationship between HL and health in the data. The lack of longitudinal associations may be due to the heterogeneous time courses and indeed the frequent occurrence of reductions in HL may be regarded as an indication of subjective misassessment at the time point T0. Adolescents may have had little experience of illness or of the health system due to their age and may exaggerate their own knowledge. Some simply do not have the expertise to assess their own health expertise. Nevertheless, longitudinal effects were observed in the T1 publication, which contradict the above argument. However, the fact that these associations were also observed in the T1 cross-section indicates that HL correlates better with itself over short intervals than over long intervals (T0-T3) (Unpublished results). In summary, the null hypotheses must be accepted here due to the observed results in the longitudinal analyses. We cannot say to what extent this result can be explained by an excessively small sample. But there is an indication in the data that the instrument for assessing HL is not optimal for the underlying age group. In future studies, it is important to consider to what extent the assessment of objective HL is more meaningful in research on adolescents than the use of an instrument that measures subjective HL, such as the HLS EU 16. In his work, In his work, Okan shows the possibility of using both subjective and objectively recorded HL in children and adolescents [50].

For a group of entrants in the first year of employment during the covid-19 pandemic, the present study shows that preventive measures are needed for high risk behaviours, overweight and poor psychological well-being. The results also show that HL is a modifiable parameter and that it is associated with health in young employees. In particular, trainees who are not in the health services or social welfare should receive more instruction on health during their training. It would perhaps be best for these sectors if the vocational colleges could assume the responsibility for this task, as training centres that are remote from patients and clients tend not to emphasise themes related to health support and prevention – apart from health and safety protection. Thus, for example, the training curricula could contain information relevant to health and emphasise personal prevention – all in addition to health and safety protection. The objective over time is then to enhance the trainees‘ HL.

### Limitations

The relative low response rate and the relatively high proportion of drop-outs increase the size of a possible selection bias in the present study. Although the design of the baseline survey included a full survey of the target group in the study region, the study sample is not representative at T3. In the drop-out analysis it has been observed, that participants with an unfavourable health behaviour had a double risk for a drop-out. So the prevalence of unfavourable health behaviour may be underestimated in the follow-up cohort. As the proportion of men at T3 is low, it is not meaningful to perform a gender-specific evaluation. Thus, it is also impossible to draw gender-specific conclusions about preventive measures. Because of the low number of cases, caution is needed when interpreting the observed prevalence. Because of the number of cases, individual sectors were combined, even when the conditions at work are different. This is particularly the base for the group office/sales/technology. In the hypothesis testing for the association between HL and health indicators, only cross-sectional indicators could be found. Thus, these findings have a lower level of evidence than associations between longitudinal data. Cohort analysis over all the time points of the survey (T0, T1, T2, T3) was not considered, due to the small number of cases, so we decided to present data for the longest follow-up period between T0 and T3 including the transition from education to the first year of work. In the longitudinal analysis there were no significant associations observed, but we cannot rule out that this was due to the small sample size. Moreover, the independent and dependent variables are from the same source, so that a bias due to common-method variance cannot be excluded. All information is based on self-reported data obtained from a questionnaire. It should also be remembered that the results were influenced by the simultaneous occurrence of the COVID-19 pandemic during the training period. This is of special relevance for employees in the health services and in health and welfare. It is possible that the observed work-related stress and demands would have been lower than during a pandemic-free period, Nevertheless, this study contains historical data, that reflects professional stress suffered by trainees during their transition to the first year of employment during the pandemic.

## Conclusions

In the present study, it was observed that there is a need for preventive action for young employees in their first year of employment that supports their health behaviour, and in particular their psychological well-being. To what extent these high prevalences are caused by selection, occupational transition or the COVID 19 pandemic, we cannot say. Generally, preventive structures in the workplace are particularly important for young entrants once they leave the vocational college, as stress at work increases during this transitional phase due to the increased responsibility. The structure of the transition to the subsequent employment phase could be improved by mentoring and health support and prevention for young employees – specifically designed for the target group. Further research on HL as a predictor for health behaviour and health should perhaps be based on the use of the long version of the HLS-EU, as this would facilitate studies on the association between HL and health behaviour. It may also be asked whether the instrument used to measure HL is suitable for trainees, and whether an objectively recorded HL in adolescents would produce more valid data than a subjective method.

## Data Availability

Data available on request due to restrictions e.g. privacy or ethical. The data presented in this study are available on request from the corresponding author. The data are not publicly available due to the fact, that this was not subject to the informed consent.
